# The Association between Dietary Inflammatory Potential and Urologic Cancers: A Meta-analysis

**DOI:** 10.1016/j.advnut.2023.09.012

**Published:** 2023-11-03

**Authors:** Ya-nan Dai, Evan Yi-Wen Yu, Maurice P. Zeegers, Anke Wesselius

**Affiliations:** 1 Department of Epidemiology, CAPHRI Care and Public Health Research Institute, School of Nutrition and Translational Research in Metabolism, Maastricht University, Maastricht, the Netherlands; 2 Key Laboratory of Environmental Medicine and Engineering of Ministry of Education, School of Public Health, Southeast University, Nanjing, China; 3 Department of Epidemiology and Biostatistics, School of Public Health, Southeast University, Nanjing, China

**Keywords:** urologic neoplasms, prostatic neoplasms, diet, inflammation, meta-analysis

## Abstract

A meta-analysis published in 2018 indicated a significant association between the dietary inflammatory index (DII) and risk of urologic cancers (UC). The number of included studies was limited, and more research has been published on this topic since then. The current study aimed to find a more precise estimate of the association between dietary inflammatory potential and risk of UC by updating the previous meta-analysis. The PubMed and Embase databases were searched between January 2015 and April 2023 to identify eligible articles. Combined relative risk (RR) and 95% confidence intervals (CI) were calculated by random-effects model to assess the association between dietary inflammatory potential and risk of UC by comparison of the highest versus the lowest category of the DII/empirical dietary inflammatory pattern (EDIP) or by using the continuous DII/EDIP score. The analysis, including 23 studies with 557,576 subjects, showed different results for UC. There was a significant association for prostate cancer among case-control studies (RR = 1.75, 95% CI: 1.34-2.28), whereas among cohort studies a null association was found (RR = 1.02, 95% CI: 0.96-1.08). For bladder cancer, a nonsignificant association was observed in both case-control (RR = 1.59, 95% CI: 0.95-2.64) and cohort studies (RR = 1.03, 95% CI: 0.86-1.24). Pooled RR from 3 case-control studies displayed a statistically significant association between the DII and risk of kidney cancer (RR = 1.27, 95% CI: 1.03-1.56). Although DII was positively associated with all types of UC, no association was found for EDIP. The present meta-analysis confirmed that an inflammatory diet has a direct effect on the development of prostate cancer and kidney cancer. Large-scale studies are needed to demonstrate the association between dietary inflammatory potential and risk of UC and provide effective nutritional advice for UC prevention.

**Protocol registration:**

The protocol was registered in the International Prospective Register of Systematic Reviews (CRD42023391204).

**Statement of Significance**

This is the first meta-analysis combining the dietary inflammatory index and the empirical dietary inflammatory pattern to observe the relationship between the diet inflammatory potential and urologic cancers.

## Introduction

With ∼2.4 million new cases diagnosed every year, prostate cancer (PC), bladder cancer (BC), and kidney cancer (KC) have been the most common urologic cancers (UC) worldwide, accounting for over 30% cancer cases and 10% cancer deaths in males [1]. Males are more prone than females to develop BC and KC [1]. A lot of research has been conducted to explore risk factors for UC to reduce the number of incidences and deaths. The most well-known risk factors are genetic factors, environmental and occupational exposures, cigarette smoking, obesity, and physical activity [[2], [3], [4]]. Besides, dietary parameters/patterns are thought to play an essential role in UC development. Research has shown that diet has the potential to reduce the incidence of all UC by 30 to 40% [5, 6]. Red meat consumption and the Western dietary pattern are thought to increase the UC risk, whereas vegetable consumption and the Mediterranean dietary pattern might lower the UC risk [4, [7], [8], [9], [10], [11], [12]]. However, according to the latest report from the World Cancer Research Fund, evidence is still scarce and generally inconsistent [13].

To understand the exact role of diets in the development of UC, it is important to understand the underlying mechanism. Evidence showed that inflammation may play a significant role in the development and progression of UC [14, 15]. It has been identified that the Mediterranean dietary pattern, which contains a high proportion of monounsaturated (MUFA) to saturated (SFA) fats and ω-3 to ω-6 polyunsaturated fatty acid (PUFAs), as well as a wealth of fruits, vegetables, legumes, and grains, has anti-inflammatory effects, whereas typical Western dietary pattern that is rich in processed meat and sugars has proinflammatory effects [16, 17]. This suggests that the inflammatory potential of diet may indeed explain the relation of these diets with UC risk. To test this hypothesis, quantitative assessable methods, such as the empirical dietary inflammatory pattern (EDIP) and the dietary inflammatory index (DII), were introduced to calculate the inflammatory potentials of personal diets and link them to UC risk, even though they differ in conception and design [18, 19]. The EDIP was developed in a United States-based prospective cohort and calculated scores for 18 food groups to assess their dietary inflammatory potential [18]. However, the DII was based on 45 dietary parameters (mainly nutrients) known to predict concentrations of 6 inflammatory markers according to peer-reviewed literature [19].

Although several previously conducted research assessed the influence of the EDIP/DII, evidence is mainly lacking and inconclusive. For PC, several case-control studies stated a strong direct association between the DII and PC risk [[20], [21], [22]], although large-scale cohort studies could not confirm this association [[23], [24], [25]]. Similarly, despite the higher DII increased BC risk in case-control studies [26, 27], cohort studies revealed nonassociations [28, 29]. Results for KC are consistent where both case-control and cohort studies showed an increased risk for people adhering to a pronounced proinflammatory diet [[30], [31], [32]]. In 2018, a meta-analysis on the influence of inflammatory indexes and UC was published, indicating that the DII was positively associated with risk of PC, KC, and BC [33]. The number of research included, however, was limited, and more studies have subsequently been published on this subject. Therefore, the current study aims to find a more precise and quantitative estimate for the association between dietary inflammatory potential and UC risk by taking into account both the DII and the EDIP to update the previously published meta-analysis.

## Methods

### Literature search strategy

A comprehensive search was performed in the electronic databases PubMed and Embase to search for eligible articles published between January 2015 and April 2023 with terms: [(dietary inflammatory potential) OR (dietary inflammatory index) OR (diet AND inflammation)) AND (urologic OR prostate OR renal OR kidney OR bladder OR urothelial) AND (cancer OR carcinoma OR neoplasm)). All research was restricted to human studies in English language. Then, all references were exported to Endnote library, and articles were manually reviewed according to the research question.

### Inclusion and exclusion criteria

**FIGURE 1:**
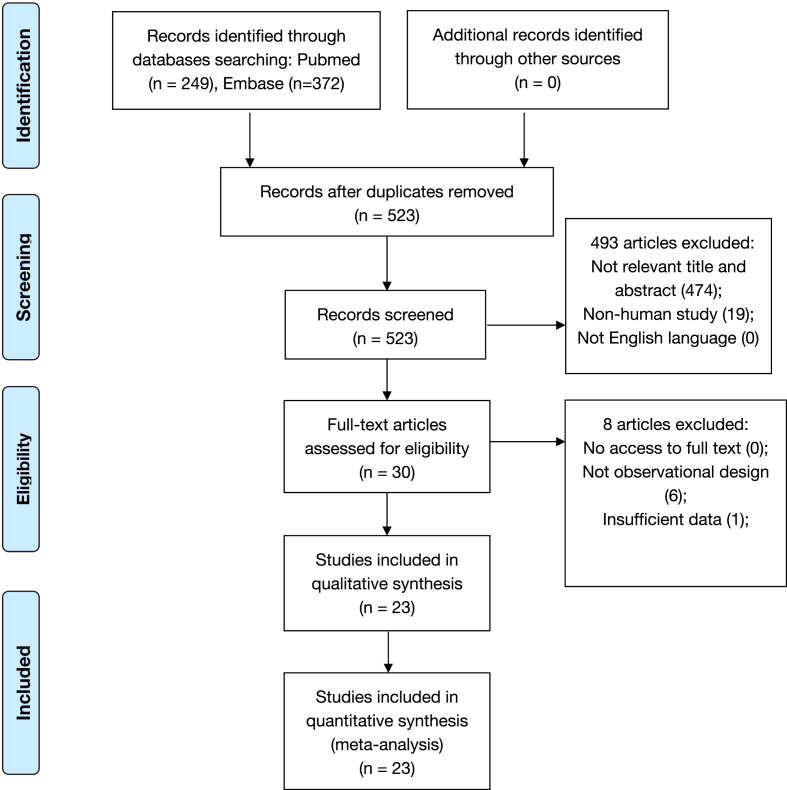
Flow diagram of literature search and selection.

Potentially relevant publications were first evaluated by screening their titles and/or abstracts, and studies meeting the eligibility criteria were retrieved. Then, all studies were assessed according to the full text whether they met the following inclusion criteria: 1) relevant topic: the association between inflammatory indexes and UC; 2) clear and definitive exposure (i.e., the DII/EDIP calculated by food frequency questionnaire), and outcome (i.e., ICD classification of PC, BC, and KC); 3) human studies; 4) observational study design. Studies were excluded based on the following criteria: 1) not English language; 2) inaccessible to full text; 3) insufficient data (without OR, RR, or HR and 95% CI for the DII/EDIP and risk of UC). Details of the study identification and selection are shown in Figure 1.

### Quality check

After study selection, the quality of all included studies was assessed by making use of the Newcastle-Ottawa quality assessment scale [34]. NOS is classified into 3 categories containing selection, comparability, and exposure/outcome, which are then divided into 8 entries. A maximum of 1 star was awarded for every high-quality item of selection and exposure/outcome, and a maximum of 2 stars could be added to the items categorized under comparability. Finally, the included studies were classified as low quality (0–3), moderate quality (4–6), and high quality (7–9) based on the number of stars.

### Data extraction

All data were extracted by YD and checked for consistency by AW. Disagreement was solved through discussion until a consensus was reached. Extracted data included the following: the first author's name, year of publication, the country where the study was performed, study duration, gender distribution, mean/median/range of age, number of cases or controls (participants for cohort studies), source of control, cancer type, scoring methods of dietary inflammatory potential, mean (SD)/median (IQR) for the DII/EDIP, the range of exposure in the highest and the lowest category, variables adjusted for in the analysis and quality score.

### Statistical analysis

Characteristics of each study and demographic details of participants were summarized using descriptive analysis. The relative risk (RR) was regarded as the common measure of the estimated associations across studies. Hazard ratios (HRs), incidence rate ratios, and odds ratios (ORs) were considered estimators of RRs. The overall association between DII/EDIP and UC risk was assessed by the comparison of the highest with the lowest category of DII/EDIP or by using the continuous DII/EDIP score. The random-effects model was applied for all analyses under the assumption that heterogeneity among studies exists and the effect sizes are different [35]. Cochran's Q test and I^2^ statistics were used to determine the presence and level of heterogeneity [36]. The Q statistic of Cochran's Q test is the weighted sum of squared differences between the study means and the fixed effect estimate. The *P* value of the Q statistic that is smaller than 0.1 indicates there is heterogeneity among studies under meta-analysis. I^2^ is interpreted as the percentage of variability in the treatment estimates that is attributable to heterogeneity between studies rather than sampling error. I^2^ > 50% indicates moderate heterogeneity [37]. Subgroup analyses based on study design and scoring methods of dietary inflammatory potential were performed for PC and BC. No subgroup analysis could be performed for KC. Potential publication bias was assessed by visually inspecting the funnel plot displaying effect size against standard error. If the funnel plot appears to be asymmetric, this may be due to small-study effects or publication bias. Begg’s test and Egger’s test were used to complement the funnel graph, of which *P* values < 0.05 reject the null hypothesis of symmetry in the funnel plot [38, 39]. The trim and fill method was used in the presence of publication bias to estimate the average effect of adjusted meta-analysis [40]. In order to test the stability of the results for UC, the leave-one-out method was used to omit one study at a time during the sensitivity analysis.

All analysis was conducted by the R package "meta" [41], and a probability < 0.05 was considered statistically significant.

### Research registry and standard guidelines

The protocol of the study was registered in the International Prospective Register of Systematic Reviews with code CRD42023391204. All procedures followed the Preferred Reporting Items for Systematic Reviews and Meta-Analyses (PRISMA) 2020 statement guidelines [42].

## Results

### Characteristics of the studies

Detailed information on the literature selection is shown in Figure 1. Eligible studies were searched in the selected databases, and a total of 523 articles were discovered. After checking duplication, relevance, and data access, 23 studies with 557,576 subjects [[20], [21], [22], [23], [24], [25], [26], [27], [28], [29], [30], [31], [32], [43], [44], [45], [46], [47], [48], [49], [50], [51], [52]] meeting the inclusion criteria were included for the pooled analysis, among which 16 studies (12 case-control, 4 cohort) reported on PC [[20], [21], [22], [23], [24], [25], 32, [43], [44], [45], [47], [48], [49], [50], [51], [52]], 6 studies (3 case-control, 3 cohort) on BC [[26], [27], [28], [29], 32, 46] and 3 studies (2 case-control, 1 cohort) on KC [[30], [31], [32]] (1 study [32] reported PC, BC, and KC at the same time). The included studies were performed on 5 continents, including North America [21, [23], [24], [25], 29, 31, 46, 52], South America [[49], [50], [51]], Europe [26, 30, 32, 44, 45, 47], Asia [20, 22, 27, 43, 48], and Oceania [28]. Other relevant information is displayed in Table 1.

**TABLE 1:** Main characteristics of the studies included in the meta-analysis

Publication Year	First Author	Country	Cancer type	Study design	Study duration	Age (mean or range)	Source of control	Gender distribution (%)	Number of participants	Assessment and level of exposure (Mean (SD) / Median (IQR))	Range of the highest and the lowest group of exposure	Variables adjusted for	Quality score
2015	Shivappa N [47]	Italy	Prostate	Case-control	1991-2002	Cases: 66 Controls: 63	Hospital based	Male (100%)	Cases: 1,294 Controls: 1,451	DII: /	> 0.49 vs. < - 1.98	Age, study center, years of education, social class, BMI, smoking status, family history of prostate cancer, and total energy intake	7
2015	Shivappa N [49]	Jamaica	Prostate	Case-control	2005-2007	Cases: 67.8 Controls: 62.0	Hospital based	Male (100%)	Cases: 229 Controls: 250	DII: -1.05 (1.11)	≥ 0.97 vs. < - 1.96	Age, total energy intake, education, body mass index, smoking status, physical activity, and family history of prostate cancer	7
2016	Vázquez-Salas RA [51]	Mexico	Prostate	Case-control	2011-2014	Cases: 67.7 Controls: 66.9	Population based	Male (100%)	Cases: 394 Controls:794	DII: Cases: 0.43 (min to max: - 4.59, 3.50) Controls: 0.52 (min to max: - 4.47, 4.51)	≥ 1.28 vs. < - 0.12	Educational level, history of PC in first-degree relatives, BMI, 2 y before the interview, PA throughout life, smoking status 5 y before the interview, history of chronic diseases and age	7
2016	Shivappa N [20]	Iran	Prostate	Case-control	/	40-78	Hospital based	Male (100%)	Cases: 50 Controls: 100	DII: /	> 0.23 vs. ≤ 0.23	Age, total energy intake, BMI, smoking status, marital status and family history of cancer, diabetes, hypertension, and cardiovascular diseases	7
2016	Graffouillère L [44]	France	Prostate	Cohort	12.6	49.2	/	Male (100%)	Cases: 123 Cohort: 2,771	DII: Cases: 0.3 (1.5) Non-cases: 0.7 (1.9)	> 1.5 vs. < - 0.98	Age, sex, intervention group, number of 24-h dietary records, BMI, height, physical activity, smoking status, educational level, energy intake without alcohol, and alcohol intake, baseline plasma PSA concentration and family history of PC in first-degree relatives	9
2016	Dugué PA [28]	Australia	Bladder	Cohort	1990–2012	Cases: 61.5 Non-cases: 54.4	/	Male (36.6%) Female (63.4%)	Cases: 379 Cohort: 37,442	DII: Cases: - 0.84 (IQR- 2.05, 0.61); Controls: - 0.98 (IQR- 2.14, 0.40)	Q5 vs. Q1 (not specify the range of each quintile)	Age, sex, country of birth, smoking, alcohol consumption, body mass index, physical activity, education, socioeconomic status, and reported intake of nonsteroidal anti-inflammatory drugs	9
2017	Shivappa N [21]	Canada	Prostate	Case-control	1997-1999	Cases: 65.1 Controls: 63.5	Hospital based	Male (100%)	Cases: 72 Controls: 302	DII: range (- 8.87 to 7.98)	> 0.68 vs. < - 0.52	Age, income, ethnicity, education, family history of a first-degree relative with prostate or breast cancer, medical history, smoking, physical activity as a teenager, energy intake, and BMI	8
2017	Shivappa N [30]	Italy	Kidney	Case-control	1992-2004	Cases: 62 Controls: 62	Hospital based	Male (64.4%) Female (35.6%)	Cases: 767 Controls: 1,534	DII: Cases: 0.13 (1.39) Controls: - 0.06 (1.38)	(0.79, 5.00) vs. (- 5.20, - 1.89)	Study center, sex, and quinquennia of age, energy intake, year of interview, education, body mass index, tobacco smoking, and family history of renal cell carcinoma	7
2017	Shivappa N [26]	Italy	Bladder	Case-control	2003-2014	Cases: 67 Controls: 66	Hospital based	Male (85.3%) Female (14.7%)	Cases: 690 Controls: 665	DII: Cases: - 0.63 (1.94) Controls: - 0.93 (2.00)	(0.42, 4.58) vs. (- 5.94, - 2.41)	Age, sex, year of interview, study center, total energy intake, education, and tobacco smoking	7
2018	Shivappa N [31]	US	Kidney	Cohort	1986-2011	55–69	/	Female (100%)	Cases:263 Cohort: 33,817	DII: - 0.87 (2.02)	> - 0.05 vs. < - 2.08	Age, BMI, smoking status, pack-years of smoking, education, HRT use, hypertension, total energy intake	8
2018	Shivappa N [48]	Iran	Prostate	Case-control	April-September 2015	Cases: 66.0 Controls: 61.4	Hospital based	Male (100%)	Cases: 60 Controls: 60	DII: Cases:1.55 (1.16) Controls: 0.93 (1.4)	> 0.96 vs. ≤ 0.96	Age, ethnicity, BMI, education, physical activity, smoking status, and use of aspirin	7
2018	Shivappa N [50]	Argentina	Prostate	Case-control	2008-2015	Cases: 72 Controls: 71	Population based	Male (100%)	Cases: 153 Controls: 309	DII: 1.47 (1.13)	> 1.96 vs. < 0.98	Age, usual BMI, energy intake and occupational exposure, and family history of cancer	9
2019	Bagheri A [43]	Iran	Prostate	Case-control	February-November 2016	Cases: 69.7 Controls: 67.9	Population based	Male (100%)	Cases: 50 Controls:150	DII: /	>0.80 vs. ≤ 0.80	Age, energy, alcohol, smoking, level of education, physical activity, family history of cancer and BMI	7
2019	Abufaraj M [29]	US	Bladder	Cohort	23	Female: 25-55 Male: 40-75	/	Male (20.8%) Female (79.2%)	Cases:1,042 Cohort: 218,074	EDIP: /	Q5 vs. Q1 (not specify the range of each quintile)	Age, smoking status, pack-years of smoking, total fluid intake, nonsteroidal anti-inflammatory and aspirin use	8
2019	Shivappa N [27]	Iran	Bladder	Case-control	/	Cases: 60 Controls: 57	Hospital based	Male (92.7%) Female (7.3%)	Cases: 56 Controls: 109	DII: - 0.12	> - 0.12 vs. ≤ - 0.12	Age, sex, BMI, physical activity, smoking status, alcohol use, and family history of cancer	7
2019	McMahon DM [23]	US	Prostate	Cohort	9.7	45-69	/	Male (100%)	Cases:2,707 Cohort: 40,161	DII: /	(-0.55, 4.89) vs. (- 6.19, ≤ - 3.36)	Age (in 5-y intervals), race, sleep, BPH, BMI, prostate cancer family history, diabetes, and smoking status.	9
2019	Hoang DV [22]	Vietnam	Prostate	Case-control	2013-2015	Cases: 68.7 Controls: 68	Population and hospital based	Male (100%)	Cases: 244 Controls: 408	DII: Cases: 0.79 (1.39) Controls: 0.20 (1.88)	≥ 1.0 vs. < - 0.59	Age, body mass index, ethanol consumption, number of children, education level, marital status, smoking habit, PC in first-degree relatives, and life-long physical activity	8
2019	Accardi G [32]	Italy	Bladder, Prostate, Kidney	Case-control	1991-2014	PC: cases: 66 controls: 63 KC: cases: 62 controls: 62 BC: cases: 67 controls: 67	Hospital based	PC: Male (100%) BC: Male (85.3%) Female (14.7%) KC: Male (64.4%) Female (35.6%)	Cases: PC (n = 1,294) BC (n = 690) KC (n = 767) Controls: 13,563	DII: PC: Cases: - 0.74 Controls: - 0.70 BC: Cases: - 0.62 Controls: - 0.93 KC: Cases: - 0.51 Controls: - 0.66	Continuous	Prostate and kidney: study center, sex (when appropriate), age, energy intake, tobacco smoking, alcohol drinking, and BMI; Bladder: study center, sex (when appropriate), age, energy intake, tobacco smoking, alcohol drinking, BMI, and diabetes	7
2019	Vidal AC [52]	US	Prostate	Case-control	2007–2018	Cases: 64 Controls: 62	Hospital based	Male (100%)	Cases: 254 Controls: 328	DII: Cases: 1.6 (−0.7, 3.5) Controls: 1.2 (−1.3, 3.7)	Q4 vs. Q1 (not specify the range of each quartile)	Age, race, BMI, smoking history, and daily caloric intake	8
2020	Luo J [46]	US	Bladder	Cohort	12.5	Male: 62.5 Female: 62.3	/	Male (48.6%) Female (51.4%)	Cases:776 Cohort: 101,721	DII Male:2.8 (2.5) Female: −4.2 (2.1)	Male: median 0.7 vs. - 5.8; Female: median - 1.3 vs. - 6.4	Randomization arm, age, race, body mass index, education, marital status, smoking status, and family history of any cancer	7
2020	Aroke D [24]	US	Prostate	Cohort	1993- 2001	62.5	/	Male (100%)	Cases:4,176 Cohort: 49,317	EDIP: /	(- 0.05, 3.98) vs. (- 5.89, < - 1.26)	Total energy intake, age at blood draw, pack-years of smoking, physical activity, sex, education, marital status, race, study center, aspirin use, ibuprofen use, nested study case-control status, family history of cancer and additionally for BMI in separate models	7
2021	Fu BC [25]	US	Prostate	Cohort	1986–2014	40–75	/	Male (100%)	Cases: 5,929 Cohort: 41,209	EDIP: /	Continuous (Per SD increase)	Age, time period, race, height, BMI, smoking status, family history of prostate cancer, PSA test in previous cycle, PSA testing in >50% of previous cycles, multivitamin use, vitamin E supplement use, alcohol intake, physical activity, and aspirin use	8
2022	Lozano-Lorca M [45]	Spain	Prostate	Case-control	2008-2013	Cases: 65.9 Controls: 66.4	Population based	Male (100%)	Cases: 928 Controls: 1,278	DII: Cases: 0.18 (1.9) Controls: 0.07 (1.9)	> 0.87 vs. ≤ - 0.95	Age, educational level, family history of PC, smoking status, body mass index, physical activity and diabetes mellitus	9

Abbreviations: BC, bladder cancer; CI, confidence interval; DII, dietary inflammatory index; EDIP, empirical dietary inflammatory pattern; HRT, hormone replacement therapy; IQR, interquartile range; KC, kidney cancer; PC, prostate cancer; PSA, prostate specific antigen; SD, standard deviation; US, the United States.

### DII/EDIP and UC Risk

#### Prostate Cancer

**FIGURE 2:**
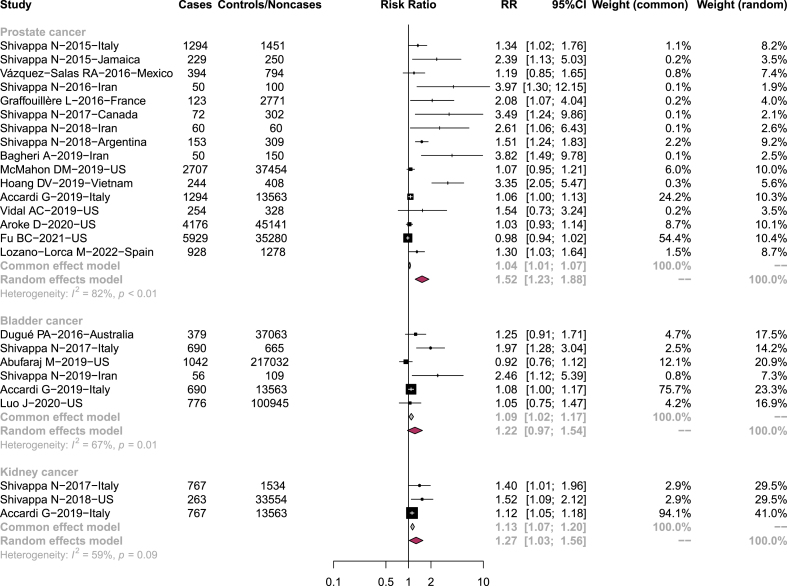
Forest plot showing RR with 95% CI for urologic cancers. Abbreviations: CI, confidence interval; RR, relative risk.

The association between the DII/EDIP and PC risk was estimated by pooling multivariable ORs/RRs/HRs from 16 studies, including over 150,000 participants. Overall, a significant relationship was found for the DII/EDIP and PC risk (RR = 1.52, 95% CI: 1.23, 1.88; Table 2, Figure 2).

**TABLE 2:** Results of association between DII/EDIP and risk of UC

Cancer type	Group	Studies (n)	RR (95% CI)	Heterogeneity	
				I^2^	*P* value of Q statistic
Prostate cancer	Overall	16	1.52 (1.23-1.88)	82%	< 0.01
	Study design				
	Case-control	12	1.75 (1.34-2.28)	81%	< 0.01
	Cohort	4	1.02 (0.96-1.08)	59%	0.06
	Dietary index				
	DII	14	1.67 (1.32-2.11)	79%	< 0.01
	EDIP	2	0.99 (0.95-1.02)	0%	0.35
Bladder cancer	Overall	6	1.22 (0.97-1.54)	67%	0.01
	Study design				
	Case-control	3	1.59 (0.95-2.64)	82%	< 0.01
	Cohort	3	1.03 (0.86-1.24)	23%	0.27
	Dietary index				
	DII	5	1.32 (1.01-1.71)	66%	0.02
	EDIP	1	0.92 (0.76-1.12)	-	-
Kidney cancer	Overall	3	1.27 (1.03-1.56)	59%	0.09

Abbreviations: CI, confidence interval; DII, dietary inflammatory index; EDIP, empirical dietary inflammatory pattern; RR, relative risk; UC, urologic cancers.

A similar elevated PC risk was observed among case-control studies (RR = 1.75, 95% CI: 1.34, 2.28), while among cohort studies, a non-association was found (RR = 1.02, 95% CI: 0.96, 1.08) (Table 2, Supplemental Figure 1).

When analyzing the different dietary indexes separately, only the DII showed an increase in PC risk (RR = 1.67, 95% CI: 1.32, 2.11), whereas no association was observed for the EDIP dietary index (RR = 0.99, 95% CI: 0.95, 1.02) (Table 2, Supplemental Figure 2).

Except for the EDIP subgroup analysis, all analyses showed moderate to high heterogeneity (P < 0.1, I^2^: 59 – 82%; Table 2, Figure 2, Supplemental Figures 1 and 2). However, omitting one study each time showed the robustness of the overall results (Supplemental Figure 3)

#### Bladder Cancer

Six studies were included to assess the relationship between the DII/EDIP and BC risk [[26], [27], [28], [29], 32, 46]. Overall, there was no statistically significant relationship between DII/EDIP and BC risk (RR = 1.22, 95% CI: 0.97, 1.54; Table 2, Figure2).

When stratified by study design, non-significant results were observed in both case-control (RR = 1.59, 95% CI: 0.95, 2.64) and cohort studies (RR = 1.03, 95% CI: 0.86, 1.24) (Table 2, Supplemental Figure 4).

Five out of 6 studies showed higher DII was associated with BC risk (RR = 1.32, 95% CI: 1.01, 1.71), whereas the EDIP did not show a significant correlation (RR = 0.92, 95% CI: 0.76, 1.12) (Table 2, Supplemental Figure 5).

Heterogeneity was observed in both the overall and the subgroup analyses with the exception of the cohort study analysis only (*P* < 0.1, I^2^: 66–82%; Table 2, Figure 2, Supplemental Figures 4 and 5).

The relationship between inflammatory dietary indexes and BC showed to be robust and in the same direction when removing one study at a time (Supplemental Figure 3).

#### Kidney Cancer

Pooled RR from 3 studies supported a statistically significant association between the DII and risk of KC (RR = 1.27, 95% CI: 1.03, 1.56). Heterogeneity I^2^ showed to be 59% (P = 0.09) (Table 2, Figure 2). This direct association remains stable during sensitivity analysis (Supplemental Figure 3).

#### Publication Bias

**FIGURE 3:**
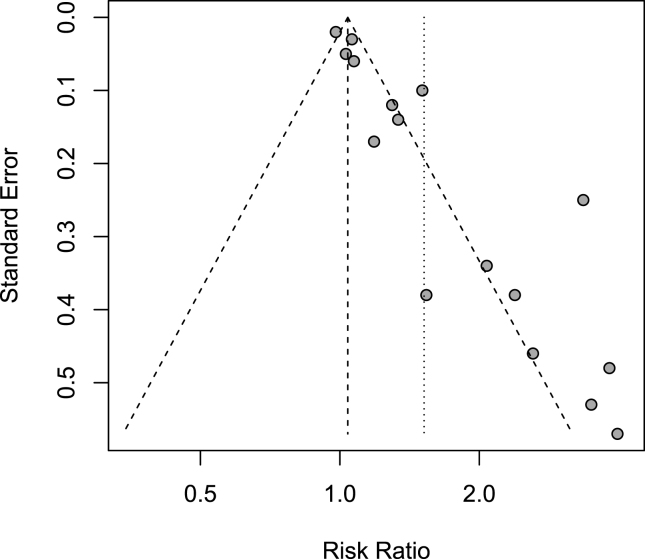
Funnel plot of the studies of prostate cancer.

**FIGURE 4:**
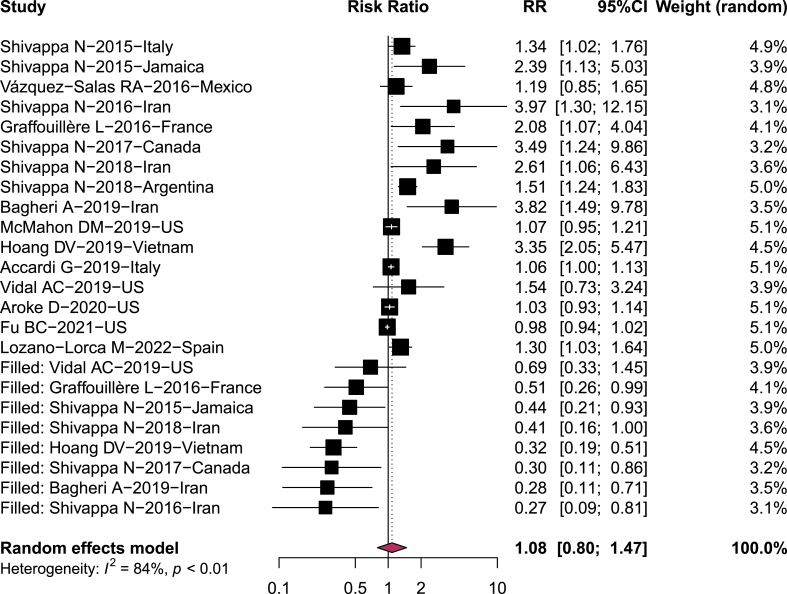
Result of trim and fill method used for detecting publication bias for prostate cancer.

Publication bias was observed for PC studies by assessing the funnel plot visually (Figure 3) and calculating the *P* value of Begg’s (*P* = 0.008) and Egger’s tests (*P* < 0.001). The overall association between the DII/EDIP and PC risk was no longer significant after adjusting for publication bias using the trim and fill method (RR = 1.08, 95% CI: 0.9, 1.37) (Figure 4). Due to the limited number of studies included, for both BC and KC publication bias could not be assessed.

## Discussion

This meta-analysis, integrating observational studies from 11 countries and 5 continents, showed an overall significant association between the DII/EDIP for PC and KC but not for BC. This association remained significant for PC among case-control studies only. In addition, while DII was positively associated with all types of UC, no association was found for EDIP.

For decades, the mechanisms leading to UC development and progression have been constantly under discussion. It becomes clear that chronic inflammation plays a major role in UC development and progression [14, 53, 54]. It is, therefore, suggested that risk factors that impact chronic inflammation, such as infections, smoking, alcohol, diets, and obesity, may be directly associated with UC development [53, 54]. The dietary indexes (the DII and the EDIP) were designed to quantitatively evaluate the inflammatory potential of personal diets on disease development and were shown to have a direct correlation with inflammatory biomarkers [24, 55].

The significant association between the DII and PC observed in the present study agrees with previously conducted meta-analyses [33, 56, 57]. This association can be explained by the fact that proinflammatory diets influence the production of proinflammatory cytokines and thus stimulate cell proliferation, resulting in DNA damage [15, 53]. A second explanation is that pronflammatory foods are usually high in calories and might, therefore, lead to obesity, which has been shown to increase PC risk [58]. Obesity promotes the release of insulin-like growth factor-1, proinflammatory cytokines, and the activation of androgen receptors and oxidative stress [58, 59]. Another explanation could be that a high-fat diet could break the balance of the gut microbiome and the release of gut bacterial metabolites, which cause short-chain fatty acids and phospholipids to enter the systemic circulation and affect distant organs [60].

The non-significant result for PC among cohort studies only could be due to the limited number of studies included in this review. Generally, cohort studies are considered to provide higher-level evidence than case-control studies. Case-control studies are prone to recall bias, which means that cases may recall their past dietary habits differently in the context of their cancer diagnosis. In addition, cases might have changed their diet before diagnosis due to early symptoms of cancer. In cohort studies, diet is assessed before the diagnosis of cancer, and recall bias and reverse causality are avoided. Furthermore, cases in case-control studies completed food frequency questionnaire after they were diagnosed with PC and were older and had lower diet quality than cases in cohort studies [61]. Harmful components (e.g., processed meat, sweetened beverages, saturated fats) in diet quality scores (e.g., American Heart Association score) are usually proinflammatory parameters counted for the DII/EDIP, which leads to a higher score of the DII/EDIP and may overestimate the association in case-control studies. Thus, case-control studies and cohort studies may produce different results. Future research should preferably be conducted in large-scale prospective cohorts to validate the findings in this review.

The present study showed that higher DII increases risk of KC. This occurs with previously published results from a meta-analysis [33]. Even though there are only 3 studies included, and this result has not been convinced by the EDIP, the significant association is supported by the potential mechanism of KC. It could be explained by the fact that proinflammatory cytokines produced by a proinflammatory diet could promote cell proliferation and transformation, resulting in DNA damage [15, 53]. Meanwhile, obesity, which is proven to be a risk factor for KC and is directly associated with the DII, could be an intermediate in the development of KC influenced by the DII [62, 63].

The present study observed a direct association between the DII and BC, in which higher adherence to the DII increased the BC risk. This is consistent with an early meta-analysis [33]. However, no effect on BC risk was observed when taking the DII and the EDIP together or by assessing the EDIP independently. The present study draws the conclusion that there is no association between higher dietary inflammatory potential and increased BC risk, taking into account the non-significant associations in both case-control and cohort studies. This finding is in line with a recently published meta-analysis consisting of 4 studies (2 case-control and 2 cohort) to investigate the association between DII and BC risk [8]. Whereas coffee, wine, and tea have no impact on BC risk, other dietary components in the DII/EDIP, such as processed meat, vegetables, and fruit, were thought to have a direct link [4]. Additionally, the Western diet and the Mediterranean diet have a substantial relationship with BC risk, suggesting that comprehensive nutritional advice is crucial for BC prevention [8]. The non-significant result for the DII/EDIP could be explained by the etiologic essentials that local chronic inflammation caused by parasite infection and catheterization, rather than systematic inflammation (circulating inflammatory cytokines), is directly associated with the development of BC [29].

The subgroup analyses in PC and BC that showed conflicting results for the DII and the EDIP might be due to the differences between them. Although both the DII and the EDIP were developed to evaluate dietary inflammatory potential, the 2 dietary indexes differ in conception and design. Whereas DII is mainly nutrient-based (i.e., 35 of its 45 components are nutrients) and assesses dietary inflammatory potential as the net effect of anti- and proinflammatory nutrients in whole diets, the EDIP is based exclusively on food groups [18, 19]. Besides, all studies applying the EDIP are cohort studies that are proven to have more moderate results compared with case-control studies.

Although the results vary across different cancer types, previous observational studies and meta-analyses have demonstrated that a proinflammatory diet may raise risk of chronic illnesses, including overall cancer [[64], [65], [66]]. The 3 urologic malignancies in the current study constitute a significant fraction of males’ cancer incidence while also having a variety of etiologies and clinical subtypes. Although obesity is frequently associated with an increased risk of UC [58, 62, 67], a diet high in calories is merely one feature of an inflammatory diet. We cannot completely rule out the possibility that other dietary elements or nutrients or their interactions with causes of cancer (for example, gender, smoking, and infection) may have a greater impact on the outcome. In the present study, the null association observed in BC supports the idea that systemic inflammation may not be as influential in the development of BC as it is in the development of PC and KC [29]. However, this hypothesis should be confirmed by further large-scale studies and experiments. Meanwhile, this result suggests researchers develop dietary patterns for specific diseases/cancers (e.g., BC) to provide rational nutritional recommendations for the population while fully understanding the disease mechanisms.

To our knowledge, this is the first review that includes both the DII and the EDIP to assess the influence of dietary inflammatory potential and UC. In addition, this review updates results, with higher statistical power, from a previously published meta-analysis [33]. However, this meta-analysis has several limitations. At first, only a limited number of studies could be included to estimate associations between the DII/EDIP and risk of BC and KC. Second, most of the included studies were case-control studies, which are shown to be prone to recall and selection bias. Third, most studies were conducted in Western countries, and results may, therefore, be restricted to certain races of the population. Fourth, the different food items and parameters used in the calculation of the DII/EDIP may lead to unfixed effects, and it may be difficult to have a unified and specific explanation and application of these 2 scoring methods, even though the random-effects model was used to assess the association. Besides, although under the assumption of heterogeneity, random-effects model was applied in all analyses, it might have resulted in an overestimate of the association due to the higher weights assigned to small studies in random-effects meta-analysis. Fifth, the non-significant result found in the trim and fill analysis of PC indicated the significant association may be inaccurate as a result of publication bias/small-study effect. Lastly, although both the DII and the EDIP were specifically designed to calculate the inflammation potential of diets because they both significantly predicted concentrations of inflammatory markers, they might both lack the ability to capture the complete complex interactions of nutrients and foods in whole diets and beverages.

## Conclusions

This is the first meta-analysis that combines the DII and the EDIP to observe the relationship between dietary inflammatory potential and UC. Results suggest that the DII/EDIP has a direct association with PC and KC but not with BC. Although the exact mechanism of inflammation in cancer is not clear, the present meta-analysis confirms that a proinflammatory diet increases risk of PC and KC. More large-scale and multicenter studies are needed to demonstrate the association between the DII/EDIP and UC and to explain the underlying mechanism pertinently in order to provide effective nutritional advice for UC prevention.

### Author contribution

The authors’ responsibilities were as follows—YD and AW designed and conducted research; YD and E.Y.W.Y analyzed data and wrote the paper; AW and M.P.Z supervised and had critical revision of the manuscript; YD and AW had primary responsibility for the final content. All authors read and approved the final manuscript.

### Conflict of interest

The authors report no conflicts of interest.

### Funding

The authors reported no funding received for this study.

### Data availability

The data sets generated during and/or analyzed during the current study are available from the corresponding author upon reasonable request.

## Declaration of interests

The authors declare that they have no known competing financial interests or personal relationships that could have appeared to influence the work reported in this paper.

## Appendix A Supplementary data

Multimedia component1 Multimedia component1
