# Supplementary material for: The Association between Dietary Inflammatory Potential and Urologic Cancers: A Meta-analysis
Source: Adv Nutr. 2023 Nov 3;15(1):100124. doi: 10.1016/j.advnut.2023.09.012 (PMC10831898; doi:10.1016/j.advnut.2023.09.012)
Supplement: Multimedia component1 [file mmc1.pdf]

Yanan Dai et al. The Association between Dietary Inflammatory Potential and Urologic Cancers: A Meta-analysis

Supplementary Material

Supplementary Figure 1 Forest plot showing RR with 95% CI for prostate cancer stratified by study design.

Supplementary Figure 2 Forest plot showing RR with 95% CI for prostate cancer stratified by dietary indexes.

Supplementary Figure 3 Results of sensitivity analyses for UC

Supplementary Figure 4 Forest plot showing RR with 95% CI for bladder cancer stratified by study design.

Supplementary Figure 5 Forest plot showing RR with 95% CI for bladder cancer stratified by dietary indexes.

Abbreviations: CI, confidence interval; RR, relative risk; UC, urologic cancers.

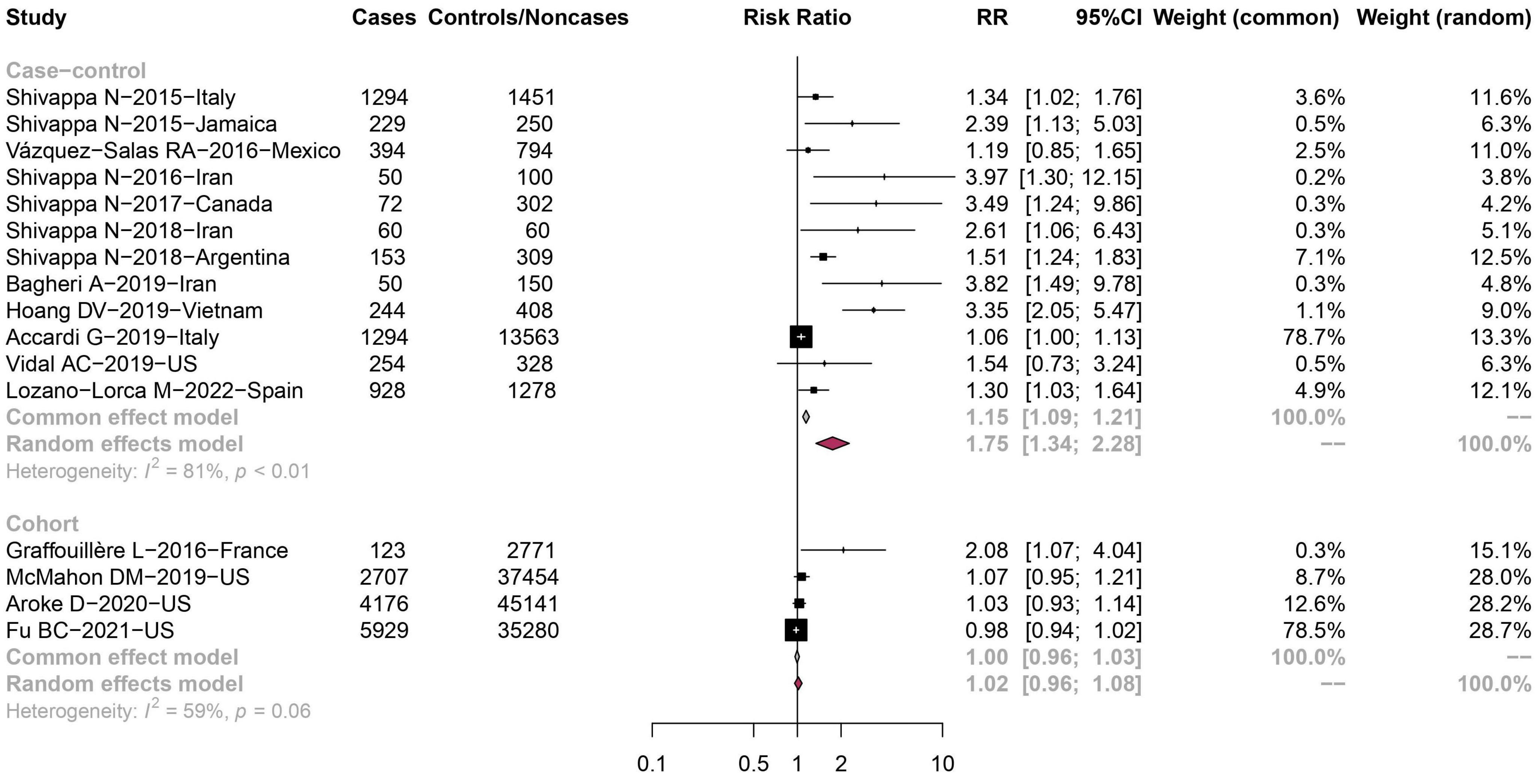

Supplementary Figure 1 Forest plot showing RR with 95% CI for prostate cancer stratified by study design.

Study	Cases	Controls/Noncases	RR	95%CI	Weight (common)	Weight (random)
**DII**						
Shivappa N−2015−Italy	1294	1451	1.34	[1.02; 1.76]	3.0%	9.7%
Shivappa N−2015−Jamaica	229	250	2.39	[1.13; 5.03]	0.4%	5.3%
Vázquez−Salas RA−2016−Mexico	394	794	1.19	[0.85; 1.65]	2.0%	9.1%
Shivappa N−2016−Iran	50	100	3.97	[1.30; 12.15]	0.2%	3.2%
Graffouillère L−2016−France	123	2771	2.08	[1.07; 4.04]	0.5%	5.9%
Shivappa N−2017−Canada	72	302	3.49	[1.24; 9.86]	0.2%	3.5%
Shivappa N−2018−Iran	60	60	2.61	[1.06; 6.43]	0.3%	4.2%
Shivappa N−2018−Argentina	153	309	1.51	[1.24; 1.83]	5.9%	10.4%
Bagheri A−2019−Iran	50	150	3.82	[1.49; 9.78]	0.3%	4.0%
McMahon DM−2019−US	2707	37454	1.07	[0.95; 1.21]	16.4%	10.9%
Hoang DV−2019−Vietnam	244	408	3.35	[2.05; 5.47]	0.9%	7.5%
Accardi G−2019−Italy	1294	13563	1.06	[1.00; 1.13]	65.4%	11.1%
Vidal AC−2019−US	254	328	1.54	[0.73; 3.24]	0.4%	5.3%
Lozano−Lorca M−2022−Spain	928	1278	1.30	[1.03; 1.64]	4.1%	10.0%
**Common effect model**			**1.14**	**[1.09; 1.19]**	**100.0%**	**--**
**Random effects model**			**1.67**	**[1.32; 2.11]**	**--**	**100.0%**
Heterogeneity: $I^2 = 79\%$, $p < 0.01$						
**EDIP**						
Aroke D−2020−US	4176	45141	1.03	[0.93; 1.14]	13.8%	49.6%
Fu BC−2021−US	5929	35280	0.98	[0.94; 1.02]	86.2%	50.4%
**Common effect model**			**0.99**	**[0.95; 1.02]**	**100.0%**	**--**
**Random effects model**			**0.99**	**[0.95; 1.02]**	**--**	**100.0%**
Heterogeneity: $I^2 = 0\%$, $p = 0.35$						

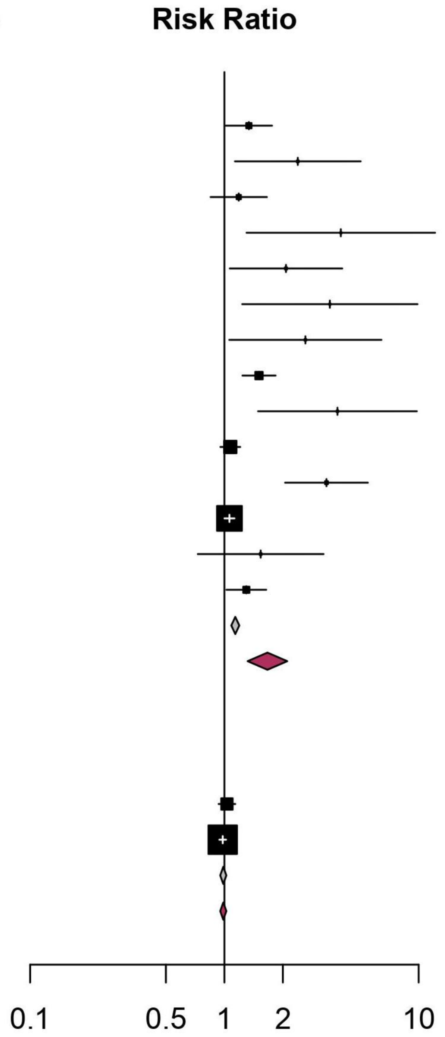

Supplementary Figure 2 Forest plot showing RR with 95% CI for prostate cancer stratified by dietary indexes.

Prostate cancer

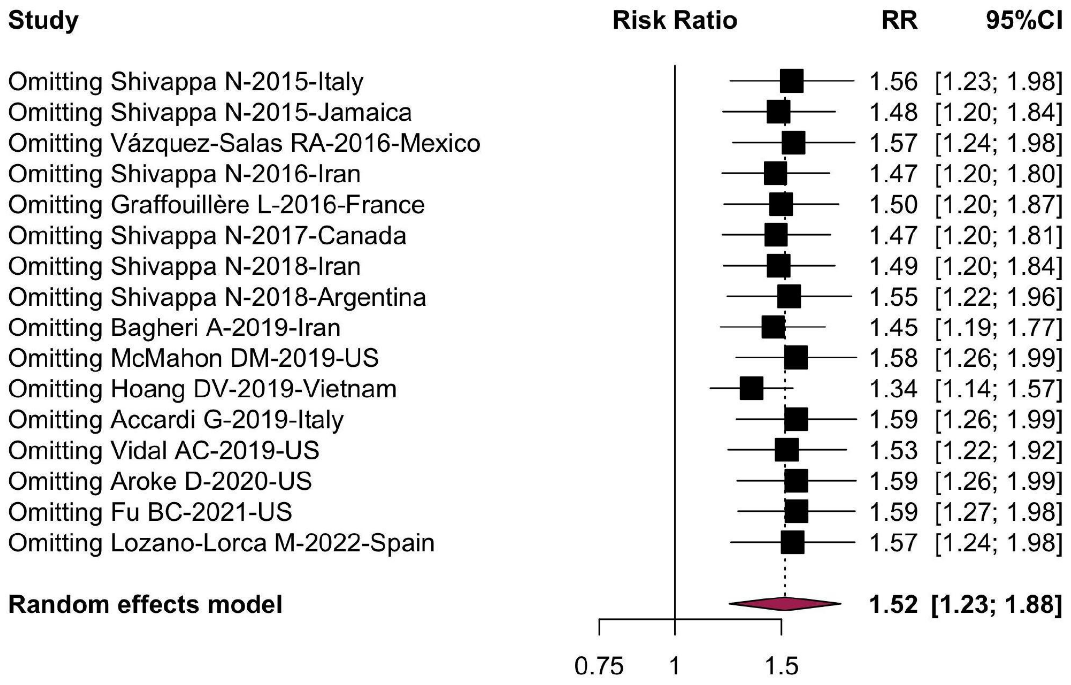

Bladder cancer

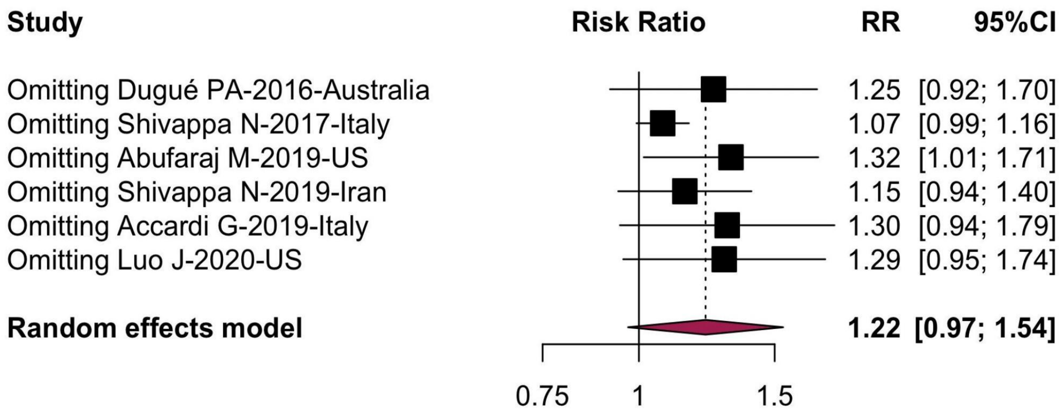

Kidney cancer

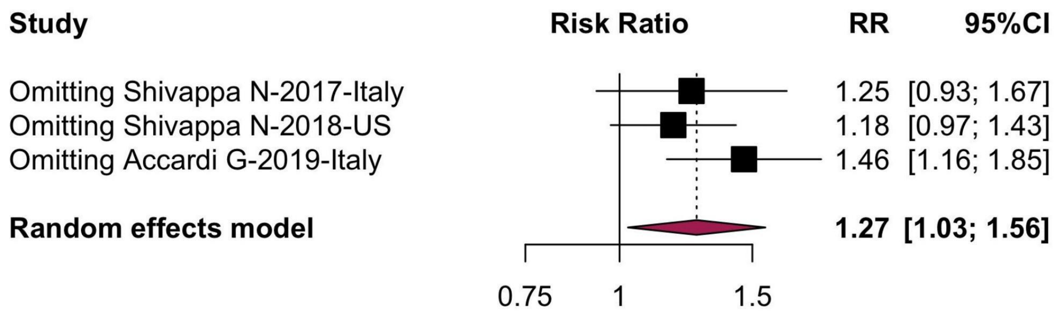

Supplementary Figure 3 Results of sensitivity analyses for UC

Study	Cases	Controls/Noncases	RR	95%CI	Weight (common)	Weight (random)
**Cohort**						
Dugué PA−2016−Australia	379	37063	1.25	[0.91; 1.71]	22.5%	31.2%
Abufaraj M−2019−US	1042	217032	0.92	[0.76; 1.12]	57.6%	38.7%
Luo J−2020−US	776	100945	1.05	[0.75; 1.47]	19.9%	30.0%
**Common effect model**			**1.01**	**[0.87; 1.18]**	**100.0%**	**--**
**Random effects model**			**1.03**	**[0.86; 1.24]**	**--**	**100.0%**
Heterogeneity: I^2 = 23%, p = 0.27						
**Case−control**						
Shivappa N−2017−Italy	690	665	1.97	[1.28; 3.04]	3.2%	30.2%
Shivappa N−2019−Iran	56	109	2.46	[1.12; 5.39]	1.0%	14.5%
Accardi G−2019−Italy	690	13563	1.08	[1.00; 1.17]	95.9%	55.2%
**Common effect model**			**1.11**	**[1.03; 1.20]**	**100.0%**	**--**
**Random effects model**			**1.59**	**[0.95; 2.64]**	**--**	**100.0%**
Heterogeneity: I^2 = 82%, p < 0.01						

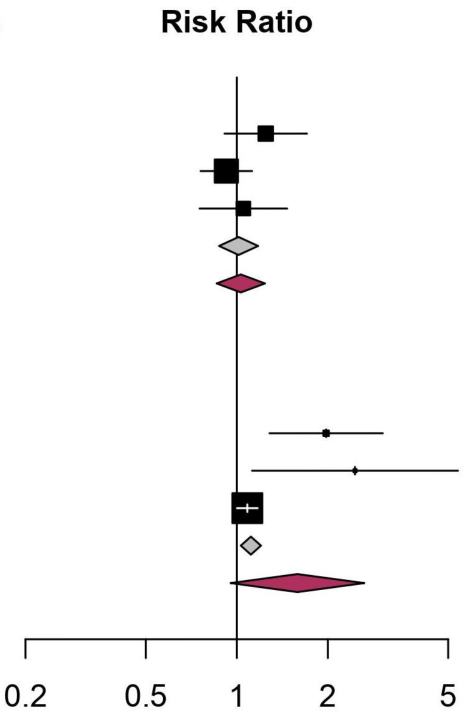

Supplementary Figure 4 Forest plot showing RR with 95% CI for bladder cancer stratified by study design.

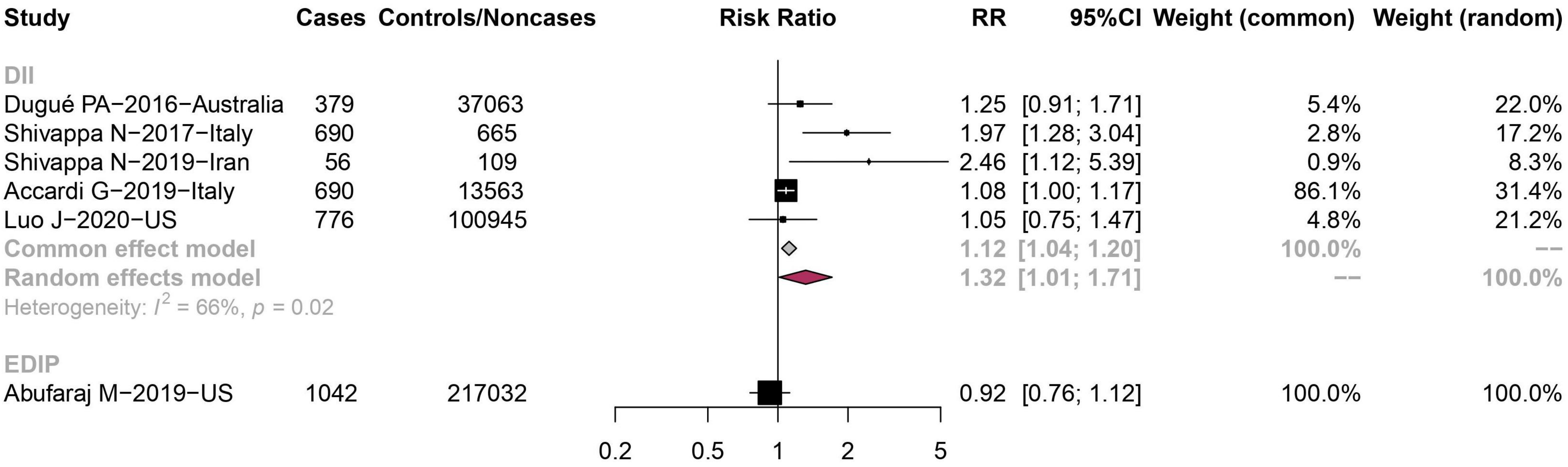

Supplementary Figure 5 Forest plot showing RR with 95% CI for bladder cancer stratified by dietary indexes.
